# NEC-zero recommendations from scoping review of evidence to prevent and foster timely recognition of necrotizing enterocolitis

**DOI:** 10.1186/s40748-017-0062-0

**Published:** 2017-12-18

**Authors:** Sheila M. Gephart, Corrine Hanson, Christine M. Wetzel, Michelle Fleiner, Erin Umberger, Laura Martin, Suma Rao, Amit Agrawal, Terri Marin, Khaver Kirmani, Megan Quinn, Jenny Quinn, Katherine M. Dudding, Tanya Clay, Jason Sauberan, Yael Eskenazi, Caroline Porter, Amy L. Msowoya, Christina Wyles, Melissa Avenado-Ruiz, Shayla Vo, Kristina M. Reber, Jennifer Duchon

**Affiliations:** 1 Robert Wood Johnson Foundation Nurse Faculty Scholar, The University of Arizona College of Nursing, PO Box 210203, Tucson, AZ 85721 USA; 2 University of Nebraska Medical Center, Omaha, NE USA; 3 Carle Hospital, Urbana, IL USA; 4 Banner Health, Cardon Children’s Medical Center, Mesa, AZ USA; 5 NEC Society, Fresno, CA USA; 6 Graham’s Foundation, Canton, GA USA; 7 Banner Health, Banner University Medical Center-Phoenix, Phoenix, AZ USA; 8 Phoenix Perinatal Associates, Mesa, AZ USA; 9 Clinical Assistant Professor and Vice-Chair, Department of Pediatrics, The University of Arizona, Tucson, AZ USA; 10 Banner Health, Thunderbird Medical Center, Glendale, AZ USA; 11 Envision Physician Services, Lawrenceville, GA USA; 12 Augusta University College of Nursing, Athens, GA USA; 13 NorthBay Medical Center, Fairfield, CA USA; 14 Hand to Hold, Dallas, TX USA; 15 Neonatal Research Institute, Sharp Mary Birch Hospital for Women and Newborns, San Diego, CA USA; 16 Stetson Hills Family Medicine, Glendale, AZ USA; 17 University of Sonora at Hermisillo, Hermosillo, Mexico; 18 Nationwide Children’s Hospital and The Ohio State Wexner Medical Center, Columbus, OH USA; 19 Columbia University, New York, NY USA

**Keywords:** Necrotizing enterocolitis, Very low birth weight, Prevention, Clinical practice guideline, Evidence-based practice, Neonatal intensive care, Infant, Nursing, Parent engagement, Translating Research into Practice Framework, NEC-zero, Practice guidelines, Scoping review

## Abstract

**Background:**

Although decades have focused on unraveling its etiology, necrotizing enterocolitis (NEC) remains a chief threat to the health of premature infants. Both modifiable and non-modifiable risk factors contribute to varying rates of disease across neonatal intensive care units (NICUs).

**Purpose:**

The purpose of this paper is to present a scoping review with two new meta-analyses, clinical recommendations, and implementation strategies to prevent and foster timely recognition of NEC.

**Methods:**

Using the Translating Research into Practice (TRIP) framework, we conducted a stakeholder-engaged scoping review to classify strength of evidence and form implementation recommendations using GRADE criteria across subgroup areas: 1) promoting human milk, 2) feeding protocols and transfusion, 3) timely recognition strategies, and 4) medication stewardship. Sub-groups answered 5 key questions, reviewed 11 position statements and 71 research reports. Meta-analyses with random effects were conducted on effects of standardized feeding protocols and donor human milk derived fortifiers on NEC.

**Results:**

Quality of evidence ranged from very low (timely recognition) to moderate (feeding protocols, prioritize human milk, limiting antibiotics and antacids). Prioritizing human milk, feeding protocols and avoiding antacids were strongly recommended. Weak recommendations (i.e. “probably do it”) for limiting antibiotics and use of a standard timely recognition approach are presented. Meta-analysis of data from infants weighing <1250 g fed donor human milk based fortifier had reduced odds of NEC compared to those fed cow’s milk based fortifier (OR = 0.36, 95% CI 0.13, 1.00; *p* = 0.05; 4 studies, *N* = 1164). Use of standardized feeding protocols for infants <1500 g reduced odds of NEC by 67% (OR = 0.33, 95% CI 0.17, 0.65, *p* = 0.001; 9 studies; *N* = 4755 infants). Parents recommended that NEC information be shared early in the NICU stay, when feedings were adjusted, or feeding intolerance occurred via print and video materials to supplement verbal instruction.

**Discussion:**

Evidence for NEC prevention is of sufficient quality to implement. Implementation that addresses system-level interventions that engage the whole team, including parents, will yield the best impact to prevent NEC and foster its timely recognition.

Neonatal complications increase the cost of prematurity 4–7 fold; [1] but complication rates vary widely among NICUs, especially for those born very low birthweight (VLBW; <1500 g) [2–4]. One of the deadliest complications is necrotizing enterocolitis (NEC), a multi-factorial acquired intestinal disease that is the primary cause of emergency neonatal surgery [5]. NEC involves systemic inflammatory activation and progresses to full intestinal necrosis when severe [6]. NEC survivors can have very long hospital stays [7], require parenteral nutrition long-term, and experience delayed neurodevelopment [8]. Preventing one case of surgical NEC can save up to $250,000 per case, and when not preventable, timely recognition is a priority [9]. Surgery is required in 20–40% of the cases; and up to 50% of those needing surgery will die [4, 6, 10].

## Background

As with many neonatal complications, NEC rates vary across NICUs [4, 11–13]. Quality improvement (QI) methods have been shown to reduce rates of NEC [14]. Central to QI is the consistent, measurable implementation of evidence into practice. In 2010, a NEC Clinical Practice Guideline published by the Cincinnati Children’s Hospital Guideline Group recommended: 1) preferential feeding of mother’s own milk (MOM), 2) providing pasteurized human donor milk (HDM) if MOM is not available, 3) using ibuprofen instead of indomethacin to close a patent ductus arteriosus (a common challenge in prematurity relating to NEC), and 4) administering antenatal steroids to mothers prior to delivery [15]. However, this guideline was not updated because of lack of a team to do so and was retired in 2015 [15]. In response, we sought to fill the gap for a NEC prevention guideline by applying a stakeholder-engaged process to conduct a scoping review and propose implementation recommendations in line with best practices to create trustworthy clinical guidelines [16–19]. To reflect the goal of preventing NEC, ultimately driving its incidence to a goal of zero, the effort was named “NEC-Zero.” As parents are the first to notice symptoms and arguably have the most to lose when NEC strikes, they participated as expert stakeholders.

## Purpose

The purpose of this paper is to present a scoping review with two new meta-analyses, clinical recommendations, and implementation strategies to prevent and foster timely recognition of NEC. All papers and position statements included in this review defined NEC as Bell’s Stage II or greater.

### Implementation science framework

**Fig. 1:**
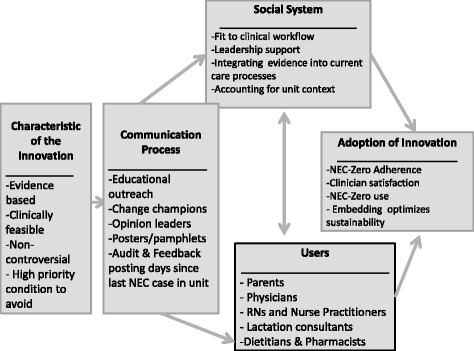
Translating research into practice framework adapted for NEC-Zero

To guide efforts, the Translating Research Into Practice (TRIP) implementation science framework was used because of its emphasis on framing evidence-based interventions in intensive care environments in partnership with stakeholders [20–23]. Building on Roger’s Diffusion of Innovation theory applied to health [24], the TRIP identifies several factors that impact adoption of evidence-based innovations in practice. Factors include 1) innovation characteristics; 2) communication processes; 3) users; and 4) the social system (see Fig. 1) [20]. The TRIP purports that to be adopted, an evidence-based intervention should be: a) better than usual care; b) compatible with clinicians’ values, c) simple, d) trialable in a low risk setting, and e) improve outcomes (process or patient-related). Figure 1 depicts how the TRIP was used to guide this stage of our process.

## Methods

### Scoping review approach

A stakeholder engaged scoping review was conducted to answer key questions about NEC prevention, timely recognition, implementation strategies and ways to engage parents [25, 26]. Six key steps are typical to scoping reviews: 1) identifying the key questions, 2) finding relevant studies, 3) selecting relevant studies to answer the questions, 4) extracting the data from the studies, 5) summarizing and reporting results, and 6) consulting stakeholders to appraise the literature, propose new resources and provide insights missing from the literature [26–28].

The group of expert stakeholders was selected in four steps. First, a national group of clinical and research experts were invited because they had published significant research and EBP improvement work around NEC. Second, a group of parents who had been impacted by NEC were recommended by the president of the Preemie Parent Alliance from the NEC Society (E.U.), Graham’s Foundation (L.M.), and Hand to Hold (T.C.). Third, a doctorally prepared Clinical Nurse Specialist engaged local stakeholders from the NICUs who intended to implement the recommendations. Finally, at the first meeting all were asked to identify expertise missing from the group, leading to more bedside nurses and a pharmacist joining. Stakeholder characteristics (*N* = 20) are portrayed in Table 1 and are referred to as “experts” from this point forward.

**Table 1:** Characteristics of NEC Working Group Experts (*N* = 20)

Characteristic	% (N) or Mean (SD)
Female	80% [16]
Years in Practice (Mean with SD)	18.6 (7.4)
Role	
Registered Nurse (Bedside NICU, Lactation Specialist, Librarian/Nurse, Neonatal Nurse Practitioner or Scientist)	45% [9]
Parent Advocate (Architect, Musician, or Information Specialist)	15% [3]
Pharmacist	5% [1]
Physician (includes Neonatologist, Medical Directors, Scientists)	30% [6]
Registered Dietician	5% [1]
Degree (Highest degree earned)	
Bachelors (B.S., B.S.N.)	20% [4]
Masters (MArch., Med., MLIS, M.S., M.P.H., or M.H.A.)	25% [5]
Doctorate (PharmD, DNP, PhD, or MD)	55% [11]
Geographical Location (United States)**	
Central	15% [3]
Eastern	20% [4]
Mountain	55% [11]
West/Pacific	10% [2]

**Eight states represented over 4 time zones

### NEC-zero description

We reviewed evidence for NEC-Zero across four evidence-based facets: 1) preferential human milk feeding; [29–36] 2) adoption of a unit-approved standardized feeding protocol; [37, 38] 3) stewarding medications particularly restricting culture-negative empiric antibiotics to <5 days [39, 40] and avoiding histamine-2 antagonists; [41–43] and 4) adopting a unit-based approach to NEC risk assessment and timely recognition [44, 45]. Withholding feedings during packed red blood cell transfusion was considered [46–48], but evidence was found to be inconclusive. We elected to exclude probiotics from this review due to controversy and lack of standardization in probiotic formulations in the US [49, 50].

Experts participated in six monthly teleconferences. To facilitate communication and ensure all voices were represented, post-meeting surveys were distributed. Meeting minutes were transcribed verbatim and shared with all participants before the next meeting. At the third meeting, subgroups were formed to allow more in-depth appraisal of literature according to each facet of NEC-Zero. Subgroup membership was distributed evenly to ensure equal representation from nursing, parents, and neonatology. The pharmacist, dietitian, and lactation consultant were specifically asked to be in certain groups (e.g. medication stewardship, feeding protocols, and human milk promotion respectively). When parent voices appeared quiet, there was follow-up after the meetings to assure time for them to contribute. Subgroups focused on one of four facets of NEC-Zero and was co-facilitated by a local stakeholder and a national expert. A recommendation template was adapted and served as an outline that assisted with searching, identifying and assessing the state of the current literature. After evaluation, rating and synthesis of the evidence was completed, the four subgroups presented their findings during an all group meeting. The research team actively facilitated the work of the subgroups.

### Selection of evidence sources

The literature search was focused to answer key questions [26, 27]. Guidelines, position statements, and studies that focused on the infant born <1500 g and were published in English were included. PubMed, CINAHL, and the Cochrane databases were searched. Targeted internet searches were applied to identify guidelines and position statements from professional organizations (e.g. American Academy of Pediatrics [AAP], American Society for Parenteral and Enteral Nutrition [ASPEN], National Association of Neonatal Nursing [NANN], Society for Breastfeeding Medicine [SBM], and the World Health Organization [WHO]). When the position was very strong, the evidence for the position was described in detail by the organization, and validated with high levels of consensus, an in-depth review of original research was deferred. If no position statement was available, systematic reviews and meta-analyses were evaluated first, followed by individual research studies if no meta-analysis or position statement was available. All participants assisted with critiquing the evidence and coming to consensus on practice recommendations [26, 27].

Experts agreed that clinicians intending to use NEC-Zero practices are likely familiar with GRADE criteria to critique quality of evidence and strength of recommendations [18, 51–53]. Meta-analyses are necessary to consider a body of evidence’s quality. In GRADE, observational studies are typically “low” quality but can be upgraded when magnitude of effects are consistent, significantly large (i.e. <0.5 or >2), confounding is accounted for or if there is evidence of a dose response. When a meta-analysis was not available, we combined study results using the Review Manager 5.3 software using random effects modeling. Recommendations are presented as “do it/don’t do it” to reflect a strong recommendation or “probably do it/probably don’t do it” to indicate a weak recommendation based on the quality of the evidence and if the quality was upgraded or downgraded (i.e. due to directness, imprecision, consistency of effects or cost balance).

## Results

### Promoting human milk feeding

The human milk subgroup addressed the evidence for human milk to prevent NEC across four categories: 1) human milk versus formula feeding; 2) human donor milk (HDM)-derived fortifier compared to cow’s milk-derived fortifier; 3) colostrum use for oral care; and 4) implementation strategies to promote human milk in the NICU.

1. Human milk versus formula feeding

#### Strength of evidence

Position Statements from NANN published in 2015 [54], The AAP in 2012 [55], AWHONN in 2014 [56], and the WHO [57] all promote human milk as the scientifically superior feeding for preterm infants. Specific health benefits for the preterm infant population including lower rates of sepsis, NEC, improved feeding tolerance, improved neurodevelopmental outcomes, lower mortality rates, more responsive immune function, lower rates of Retinopathy of Prematurity and fewer hospitalizations in the first year post-NICU discharge compared to formula feeding. [55] A meta-analysis concluded that if the preterm or low birth weight infant cannot have access to their mother’s own milk (MOM), meta-analyses demonstrate that pasteurized HDM demonstrates protection from NEC versus the use of preterm or term formula [58]. Prioritizing the use of MOM over DHM is important because MOM is more bioactive than DHM, contains more immune-supporting human milk oligosaccharides [59] and is more protective against NEC [60].

#### Recommendations

We agree with the AAP position that all preterm infants should receive human milk and that if MOM is not available, pasteurized DHM is preferred to formula [55]. (High quality, do it).

2. Use of donor human milk (DHM)-based fortifier versus Cow’s milk-based fortifier

#### Strength of evidence

**Fig. 2:**
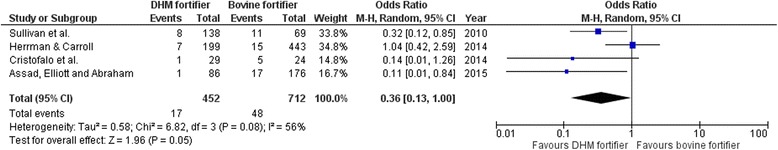
Pooled effects of donor human milk-based fortifier compared to cow’s milk-based fortifier on odds of NEC

The AAP recommends that human milk be fortified for infants born less than 1500 g [55]. Fortification can be accomplished with adding cow’s milk-based fortifier or DHM-based fortifier to human milk. Some refer to a diet that includes MOM, DHM if MOM is unavailable, and DHM-based fortifier as an “exclusive human milk diet.” Four studies have evaluated the difference in NEC (defined as Bell’s stage II or greater) between the two types of fortified diets [30, 61–63]. When results were pooled from two RCTs [30, 61], lower risks of death, NEC, NEC requiring surgery, and sepsis in infants less than 1250 g was shown with risks rising incrementally as the percentage of cow’s milk in an infant’s diet increases [64]. Since 2014 when the pooled analysis was published, two more cohort studies have been published [62, 63]. We applied a random effects model to conduct a meta-analysis of the four studies for infants weighing <1250 g at birth (*N* = 1164) and show that infants fed with DHM-based fortifier had approximately 64% lower odds of NEC compared to those fed with bovine based fortifiers (OR = 0.36, 95% CI 0.13, 1.00, *p* = 0.05; Fig. 2). Highest protection of DHM-based fortifier was shown in units with high rates of NEC and cost-savings from NEC avoidance may be low if the baseline NEC rate is low. One limitation of the evidence is that it focused on the infant <1250 g and the effect estimate included one. More studies are needed in NICUs with pre-treatment NEC rates that are typical for most NICUs vs. those in the literature in higher rate NEC NICUs. No studies have shown adverse effects of using human milk based fortifiers although adequate growth should be monitored [65].

#### Recommendations

The subgroup recommends the use of DHM-based fortifier over bovine based fortifier (Moderate quality; probably do it) with prioritized MOM with DHM if MOM is not available. In units with a low baseline incidence of NEC, the cost of DHM-based fortifier may show lower cost-effectiveness compared to those with a high baseline incidence. Greatest effects of DHM-derived fortifier to reduce NEC are shown in units with high baseline NEC incidence.

3. Colostrum as oral immune therapy

#### Strength of evidence

The use of colostrum for oral care to provide immune therapy in preterm infants was next addressed. Evidence reviewed consisted of 1) a narrative review; [66] 2) three randomized control trials; [33, 67, 68] 3) two cohort studies; [36, 69] 4) a qualitative study; [70] 5) two pilot studies; [32, 35] and 6) a position statement. [54] The studies were typically single site and underpowered to answer questions related to NEC outcomes. However, many of the studies support the safety and feasibility of early colostrum oral care in extremely-low- and very-low-birthweight infants, [32, 33, 35, 36, 68, 69] and specifically in intubated babies [68, 69]. Use of colostrum for oral care impacted other important neonatal outcomes such as reaching full feeding volume earlier [33], earlier initiation of enteral feedings and better weight gain at 36 weeks corrected gestational age, [36] boosts in immune markers suggesting immune-protection, [68] and a reduction in the length of stay [67]. At least one multi-center RCT is in progress and powered to detect differences in late-onset sepsis, NEC and death outcomes [71]. In one qualitative descriptive study of mothers with infants who had congenital diaphragmatic hernia, strong themes emerged that mothers and family members found meaning in providing colostrum oral care emphasizing that it encouraged them to continue pumping their milk [70]. Although using colostrum for oral care is shown as very low-risk, it is not clear from the studies what the optimal duration or dose is. In the studies reviewed, colostrum oral care was typically started by 48 h of age and continued for 2–5 days. No clinical studies support using DHM for oral care at this time because none are available.

#### Recommendations

Based upon immune boosting and benefits to promote mother’s milk supply, colostrum for oral care is recommended, although its direct effect on NEC has not been shown (Low quality, probably do it).

4. Implementation strategies to promote human milk in the NICU

#### Strength of evidence

There are a multitude of articles and position statements that unanimously support providing human milk to all infants, but particularly emphasizing the health benefits for infants born early. A recent cost analysis estimated implementation gap burden of failing to provide premature infants with adequate volumes of human milk equates to 1.5 billion dollars annually in the US alone [72]. Implementation guidance is provided by NANN to use a programmatic approach, recommending Spatz’s Ten Steps to promote human milk in the NICU [54, 73]. We critiqued the evidence about best implementation strategies to support mothers of premature infants to provide human milk. While the overall effectiveness of human milk promotion programs was shown, few studies were focused on the implementation science behind them. Overall, they showed that to a whole-team approach is needed that systematically and consistently engages mothers with adequate lactation education, pumping support and assessment of adequate milk supply.

#### Exemplar programs and related resources

Three implementation programs to promote human milk in the NICU were selected as exemplars and reviewed by the subgroup. Each program has videos, education materials, and education content for staff. Measurement of program success have shown by increased breast pumping initiation rates, longer duration of pumping or breastfeeding, higher volumes of milk the infant received, and demonstration that the infant was still receiving human milk at discharge from the NICU [74, 75, 73]. These programs consistently engage parents, moving them from bedside bystanders and validating their essential role on the healthcare team.

#### Recommendations

The subgroup recommends several steps be adopted to initiate organization-level, provider- level and patient level change to promote human milk and that using a program supports this multi-layered implementation (Table 2) [60, 73, 74]. The strength of these recommendations is based on lower levels of evidence that specifically studied implementation (Low quality, probably do it). More research is needed using high quality designs to assess effective implementation strategies on human milk outcomes.

**Table 2:** Recommendations and Implementation strategies for NEC Prevention

Promoting Human Milk	
Clinical Recommendations and GRADE	Implementation Strategies
1. Mom’s own milk (MOM) is the preferred first line nutrition for preterm infants (except for in cases where it is contraindicated). If no MOM is available, donor human milk (DHM) is preferred over formula. [High quality, do it] 2. DHM-based fortifier is preferred over bovine based fortifier. Benefits of human milk based fortifiers outweigh the risks. Can be cost-effective for the healthcare system, with greater cost savings likely in higher rate NICUs. Impact of human milk-based fortifier on growth is inconsistent across studies and growth should be monitored carefully [Moderate quality, probably do it] 3. There is documented benefit from using colostrum for oral care to boost immune response and to encourage mothers to sustain milk production. (Low quality, probably do it).	Adopt a hospital-based policy to support breastfeeding and providing human milk. Provide education by OBs and Neonatologists when preterm delivery is anticipated about the importance of human milk emphasizing immune as well as nutritional benefit. Reiterate importance of breastmilk for preemies in a parent handbook or pamphlet, translated into commonly spoken languages and written in simple terms. Support initiation of pumping within 6 h after delivery and offer pumping at the bedside when possible. Provide lactation specialist support early (<24 h) and consistently through the stay. Educate staff (e.g. post-partum RNs, NICU RNs, Residents) using diverse training tools. Use huddles to remind staff about human milk education and goals. Provide pumps in the hospital and resources to rent pumps at home. Track initiation of pumping and milk volumes. Use colostrum for oral care. Facilitate regular skin to skin care (aka “kangaroo care”). Offer peer lactation support. Promote non-nutritive breast feeding when the infant is stable. Encourage nutritive breast feeding when appropriate and recommend breast before bottle when possible. Create a breast feeding plan for discharge.
Standardized Feeding Protocols	
Clinical Recommendations and GRADE	Implementation Strategies
1. Adopt a unit-approved standardized feeding protocol to reduce inter-provider variation. [Moderate quality, do it]. 2. A multi-disciplinary team should be involved in creating, implementing and monitoring adherence to the protocol.	Consider “Feeding rounds” as a way to audit and feedback on compliance with the feeding protocol. Track initiation of feeds. Track advancement of feeds. Track fortification of feedings. Track growth. Formalize criteria for identifying and managing feeding intolerance. Tie feeding protocol to competencies and ongoing staff education.
Timely Recognition of NEC	
Clinical Recommendations and GRADE	Implementation Strategies
1. Early recognition tools can be beneficial in patient safety efforts. Validated tools have been shown to differentiate between infants who get NEC compared to those who do not. [Very low evidence, probably do it]	Consider risk tool to use at the unit level (e.g. GutCheckNEC, NeoNEEDS or eNEC). Use a structured communication script (e.g. Situation-Background-Assessment-Recommendation; SBAR method) to communicate when NEC is suspected and to focus assessment. Educate parents about warning signs of NEC and preventive measures verbally and with printed materials (e.g. pamphlets) written in a way parents can easily understand. Optimal timing for this education is when initiating, advancing, or adding fortification to feeding. Use medically accurate terminology when communicating with parents (e.g. “necrotizing enterocolitis” vs. “tummy problems”, etc.) Communicate baby’s risk factors to parents and emphasize why human milk is important to help prevent NEC and that they play an important role in NEC prevention. Empower parents and nurses to speak up when concerned.
Medication stewardship	
Clinical Recommendations and GRADE	Implementation Strategies
1. Avoid use of H2 blockers within the first 120 days of life (enteral or parenteral) [Moderate quality, don’t do it] 2. Restrict empiric antibiotic use to 4 days or less for infants without positive blood cultures or clinical suspicion of infection [Moderate quality, don’t do it]	Specify, adopt and automate prescribing guidelines for antibiotics that require a specific number of doses to be ordered. Adopt electronic alerts that warn the clinician that an H2 blocker is ordered and that it increases the risk for NEC. Communicate at handoffs about the date and time antibiotics should be stopped. Collaborate with pharmacists and integrate electronic alerts into electronic health record to remind clinicians to stop unnecessary antibiotics. Educate hospital personnel (e.g. neonatology, nursing, physician trainees) on recently published guidelines. Participate in antibiotic stewardship and regional collaborative organizations in multidisciplinary teams. Evaluate change by measuring the adherence to protocol and the number and % of infants who received prolonged antibiotics or H2 blockers. Create and share a report on findings within the local NICU. Give feedback to clinicians on their adherence to the medication stewardship guidelines in a way that is timely, individualized, not punitive, and customizable.

#### Engaging parents

Parent subgroup members advised that earlier education about the importance of human milk to help them make an informed decision about providing human milk be given and specific guidance on how to bring in, maintain and monitor milk supply shared. Concerns were raised about delays to initiate pumping, lack of printed education, and materials that did not show women from diverse communities (e.g. African American or Hispanic) breastfeeding. When providing human milk for their vulnerable infant was being presented as a “choice” rather than a necessary medical treatment, they experienced angst at receiving mixed messages from the healthcare team about the importance of human milk. They recommend that lactation education is started before delivery and that they are shown how to set up the breast pump at that time. Fathers were not asked their perspective but mothers were emphatic that supportive partners are critical to the “human milk producing team.” In sum, parents recommended 3 key strategies to support them to provide human milk: 1) early and often skin to skin holding, 2) early pumping (i.e. within the first 6 h, preferably within 2 h), and 3) access to lactation support regardless of intention to breastfeed.

### Standardized feeding protocols

#### *Strength of Evidence*

**Fig. 3:**
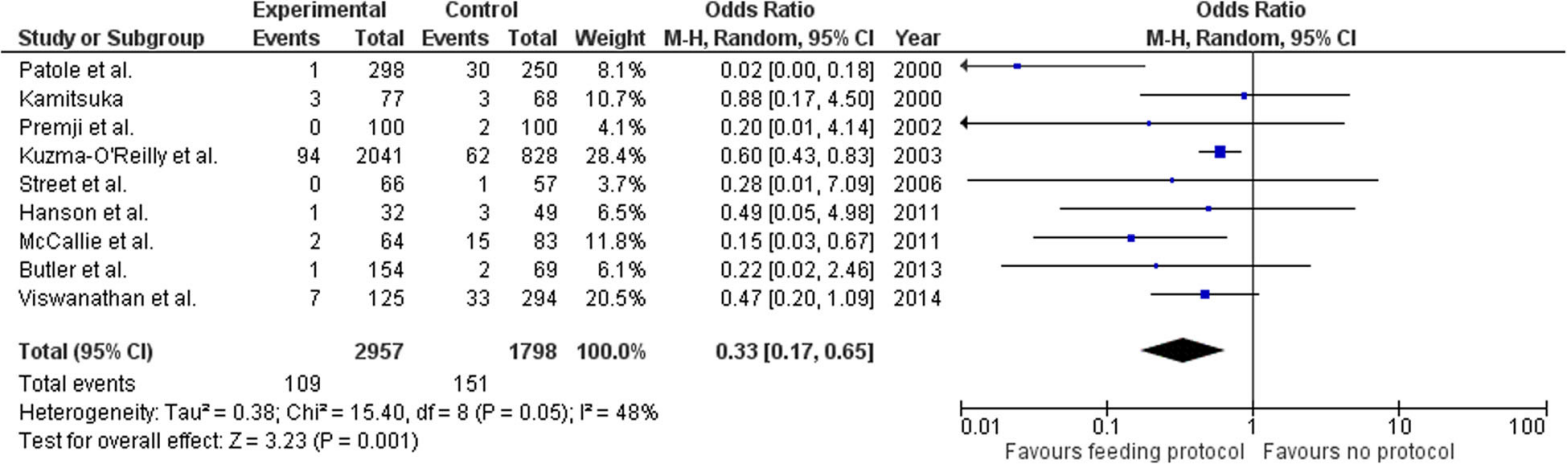
Pooled effects of standardized feeding protocol on odds of NEC

Standardized feeding protocols (SFPs) address a consistent approach to the: 1) initiation and duration of trophic feeding; [76–79] 2) advancement and fortification of feeding; [77, 80] 3) criteria to stop and specifying how to re-start feedings once held; 4) identification and handling of feeding intolerance; [37, 81, 82] and 5) preferred feeding substance. Patole and deKlerk conducted a meta-analysis in 2005 of 6 observational studies showing reduced risk for NEC of up to 87% for infants <2500 g, when a feeding protocol is in place, even when formula was used within the protocol [38]. Studies were heterogeneous (*p* < 0.001) but when looking at studies similar to each other, pooled risk ratios were more modest (RR 0.71, 95% CI, 0.52 to 0.97) conferring about a 29% reduction in risk for NEC for infants <1500 g. [38] In 2016, our team reviewed papers published since the meta-analysis. When SFPs are used, studies consistently showed lower or unchanged NEC rates [81–86], with some also showing reduced late onset sepsis [83], and fewer days of parenteral nutrition [81, 85]. Weight gain improved after implementing SFPs in some studies [81, 83, 85], and one study showed less occurrence of bronchopulmonary dysplasia [81]. No study showed an increase in NEC rates or any other adverse events. No studies used randomized controlled designs. To pool studies published since 2005 with the Patole and deKlerk meta-analysis, we applied a random effects model combining data from 9 observational studies (*N* = 4755 infants <1500 g) [81, 83–85, 87–91]. Figure 3 shows an overall reduced odds of NEC by 67% (OR = 0.33, 95% CI 0.17, 0.65, *p* = 0.001) with moderate heterogeneity across studies (I [2] = 48%) when SFPs are used. We limited the counts used in this meta-analysis to those infants <1500 g.

Evidence was not conclusive, and more research is needed to identify best strategies for holding feedings when an infant is critically ill (e.g. very hypotensive or receiving prophylaxis for intraventricular hemorrhage with indomethacin), or during packed red blood cell transfusion. Experts did not examine whether continuous or bolus feeding is better. Although further randomized, controlled trials would be useful to increase the quality of evidence supporting SFPs, this type of study will be difficult to justify and conduct in centers that already have SFP in place. Pragmatic multi-site clinical trials that compare effectiveness of one SFP to another using randomized experimental designs could be useful.

#### Recommendations

We recommend the use of a unit-approved standardized feeding protocol based on the magnitude of their effects to reduce NEC, their low cost, and low risk (Moderate quality, do it). Details of the protocol itself do not appear as important as reducing inter-provider variation [38, 92]. A SFP should be adopted at the NICU level (i.e. all providers agree to its components) and address: 1) when to initiate and how long to give trophic feeding (typically 10–20 mL/kg/day for 48–72 h); 2) schedules to advance and then fortify feeding (e.g. typically increasing calories by adding fortifier at 22 kcal/oz. after reaching 80–100 mL/kg/day and increasing to 24 kcal/oz. after reaching 120 mL/kg/day); 3) criteria for stopping and restarting feeding (e.g. if a unit decides to hold feeding during blood transfusion, indomethacin, etc.); 4) prioritized fresh human milk (MOM 1st, DHM 2nd, preterm formula 3rd); and 5) criteria to identify, and manage feeding intolerance. Best outcomes across studies appear to be shown when a multi-disciplinary team is involved in creating, implementing and monitoring adherence to the protocol. Guidance on components of effective protocols has been published by ASPEN [77] and the California Perinatal Quality Collaborative [83, 93]. Based on group consensus and recommendations from published papers, [81, 83, 91, 94] several SFP implementation strategies are recommended (see Table 2).

#### Timely NEC recognition

Beginning signs of NEC occur at approximately 2–4 weeks of age, often when infants have experienced multiple encounters with multiple clinicians [10, 95]. Information relevant to recognizing NEC is contained in many places in the medical record, making it a challenge for clinicians to integrate into their assessments. Nurses and parents often recognize NEC first, but when symptoms are mild or non-specific, treatment delays can occur if communication is unclear or when symptoms are not considered in the context of NEC risk factors [45]. The timely recognition subgroup evaluated biomarkers, bedside monitoring techniques, and information-based tools to assess NEC risk and addressed ways to engage families in the process. Although timely recognition was the goal of the subgroup, based on review of evidence the focus shifted to what was possible to support *timely* recognition because it is not clear from the evidence to what extent early recognition is possible. In other inflammatory disease processes, the longer the time to treatment the more severe the illness becomes and the more difficult it is to treat it.

#### Strength of evidence

Given the purpose of identifying strategies to adopt in clinical practice, this group evaluated what is currently *most available* and *feasible to implement*. Based on a focused literature search in 2015, several promising noninvasive biomarkers were identified but none appeared widely available for clinical use outside of a research protocol [96, 97]. Bedside monitoring tools to evaluate intestinal perfusion changes did not include guidelines to make them readily implementable [98–100]. Risk scores found in 2015 to promote NEC risk awareness include eNEC™ [45, 101], NeoNEEDS [102], and GutCheck^NEC^ [44, 45]. No tool showed perfect prediction but all showed promise to differentiate between infants who got NEC compared to those who did not. Implementation of NeoNEEDS showed a shift towards fewer severe NEC cases as more “suspected NEC” was identified. eNEC has been clinically tested in a QI project with high inter-rater agreement and positive impact on increasing knowledge of nurses about NEC risk factors [45, 101]. GutCheck^NEC^ was tested with the most infants and showed robust prediction for NEC leading to surgery (AUC = 0.84, 95% CI 0.82–0.84) or death (AUC = 0.83, 95% CI 0.81–0.85) but its ability to discriminate medical NEC was marginal [44, 45].

Much discussion in this group centered on communication strategies and ways to engage parents as timely recognition partners. Review of evidence on communication strategies that support patient safety and rescue protocols showed that structuring communication (e.g. at the change of shift or handoff of care from clinician to clinician or when a nurse calls a physician or NNP) can support clarity and reduce communication failures [103–105]. An international study of families’ experiences around NEC communication conducted by the NEC Society identified that information about NEC was most often shared verbally and primarily at the time of diagnosis [106]. Very few parents received anticipatory guidance about warning signs to watch for, preventive strategies they could take, or how different treatments may increase their risk. Parents expressed the great need to be believed when they saw their child “not acting right” and bore the guilt of not advocating for their baby when the outcomes were poor [106]. The subgroup agreed that it would be helpful to have a tool to share with parents but did not want to scare parents or expose them to unnecessary stress and worry. In contrast, parents firmly believed that clinicians should share critical information with parents instead of avoiding doing so for fear of scaring them.

### Recommendations

Timely recognition tools can support consistent communication and are shown beneficial in patient safety efforts. Validated tools have been shown to differentiate between infants who get NEC compared to those who do not. Benefits of using them is likely to outweigh risks although more research is needed (Very low evidence, probably do it) [44, 101, 102]. Structuring communication when NEC is suspected in tandem with adopting a risk score was the most implementation-ready strategy to support timely recognition. Such a risk scoring system can also be used to educate staff about NEC risk factors and cue attention to times (e.g. day of life and contexts) when NEC is most prevalent. We recommend that tools like GutCheck^NEC^, eNEC or NeoNEEDS be coupled with a focused assessment tool that is organized using a Situation-Background-Assessment-Recommendation (SBAR) format. More research is needed on ways to implement biomarkers and bedside monitoring (e.g. NIRS) into routine practice.

#### Engaging parents

Parents should be empowered to speak up when they think their baby is not acting right for several reasons including that they know their infant best, they are the most consistent bedside caregiver, and they have the most to lose. Further, parents should be educated on warning signs that signal a change. The group discussed at length the best timing to discuss the symptoms of NEC with parents. The consensus was that discussions could occur when human milk education is given, when feedings are started, advanced, and changed. If signs of feeding intolerance arise, discussions about warning signs of NEC can be addressed. Throughout the discussions, emphasis can be placed on what parents can do to help prevent NEC (e.g. provide human milk) and what the team is doing to watch for it. When parents raise concern that their infant is not acting right, including parent concern as part of a focused assessment tool can support nurse to provider communication. As part of this subgroup’s activities, a website was developed to share tools created to engage parents including videos, pamphlets (also in Spanish) and links to diverse family support resources (see http://neczero.nursing.arizona.edu/). A focused assessment tool that combines GutCheck^NEC^ with an SBAR script to support communication when concerns arise is also available at this website.

### Medication stewardship

This subgroup worked to address questions related to prolonged antibiotic therapy, barriers to limiting antibiotic therapy, and implementation and monitoring of antibiotic and H2 blocker stewardship to prevent NEC.

#### Strength of evidence

Three multi-site observational studies in the US (23 NICUs, combined *N* = 4716 infants) have addressed the role of an extended initial course of antibiotics on risk for NEC [39, 40, 107]. Cotten and colleagues evaluated a prolonged course of antibiotics as a measure of risk associated with each additional antibiotic day with an exclusive focus on infants born weighing <1000 g [39]. The other 2 studies included infants <1500 g with one applying a case-control approach in a single NICU [40], and the second evaluating a cohort of 3 NICUs from a state network over 4 years [107]. Two excluded studies compared those who received no antibiotics compared to those who did for early empiric therapy but did not evaluate the impact of a *prolonged course of* antibiotics [108, 109]. Consistently, all 3 studies showed an increasing odds of NEC or death after 4 days of empiric antibiotics when blood cultures were negative [39, 40, 107]. The decision to restrict antibiotics can address markers of inflammation as well as the presence of a negative blood culture because not all infected babies will have positive blood cultures [110].

Evidence addressed risk of NEC with H2 antagonists came from a systematic review with meta-analysis of 2 cohort studies (*N* = 11,346, <1500 g) [111]. When pooling the two studies [41, 42], they found significant heterogeneity (I [2] = 73%) but an increased odds of NEC when gastric acid inhibitors (proton pump inhibitors) or H2 receptor blockers (ranitidine, famotidine or cimetidine) were given parenterally or enterally before NEC (OR = 1.78, 95% CI 1.4, 2.27, *p* < 0.00001). They judged the risk of bias to be low to moderate using the Newcastle-Ottawa scale.

#### Recommendations

The subgroup recommends judicious antibiotic use to 4 days or less for infants without positive blood cultures or clinical suspicion of infection (Moderate quality, don’t exceed 4 days of antibiotics unless highly suspected or proven infection) [39, 40, 107, 112]. When ordering empiric antibiotics, a stop-date or a specific number of doses should be ordered. Use of H2 blockers increases the odds of NEC and sepsis [111] and should be avoided (Moderate quality, don’t do it). This group identified ampicillin and gentamicin as the first choice treatment for early onset newborn sepsis [113], restricting higher order cephalosporin use (e.g. cefotaxime and cefepime) to select cases [112]. Preferred duration of therapy was for 48 h to rule out infection based on evidence that showed each additional antibiotic day confers an increased risk of NEC or death [39, 40]. Individual groups may choose to adopt restrictions from longer than 48 h but these should be limited to less than 5 days.

#### Implementation strategies

The group identified several barriers to implementing a restrictive approach to antibiotics and H2 blockers. These included a concerning clinical presentation, such as respiratory distress, cardiovascular instability, or abnormal lab indices. [114] Using a sepsis calculator may be useful, however current tools are only validated for infants >34 weeks [115, 116]. Clinicians may not be knowledgeable about current recommendations so their practice may not reflect current best evidence. In busy units where attention is focused on cardio-respiratory crises; it is possible that discontinuing antibiotics (a routine task) may be overlooked. If between-shift handoffs are unstandardized, failing to communicate a plan to discontinue antibiotics is more likely. To overcome these barriers, several implementation strategies are described in Table 2.

## Discussion

NEC remains a chief threat to the survival and health of premature infants in spite of the evidence available to reduce its incidence. Implementation guidance, toolkits, and strategies to engage parents are needed to forward improvement efforts. Prioritizing a human milk diet was best supported by evidence, position statements, and stakeholder input. While a specific feeding protocol could not be recommended, the group agreed that using a feeding protocol is evidence-based. Two new meta-analyses conducted as part of this scoping review supported the protective effect of a DHM-based fortifier and feeding protocols to reduce odds of NEC. Avoiding >4 days of antibiotics for the initial empiric course after birth and avoiding any exposure to histamine-2 antagonist medications was recommended.

This scoping review engaged expert stakeholders to review evidence focused on answering key questions and make recommendations to prevent and support timely recognition of necrotizing enterocolitis. Four subgroups reviewed evidence from 11 position statements and 71 research publications. Discussion about the evidence yielded 29 actionable recommendations and guidance on implementation strategies. This approach engaging national experts with local clinicians and parent representatives was consistent with recommendations for designing trustworthy clinical practice guidelines laid out by the National Academy of Medicine and others [16–19]. A geographically diverse expert group yielded a real-world approach to implementation of NEC prevention strategies. Evidence was strongest for promoting a human milk diet, use of a unit-adopted standardized feeding protocol and limiting exposures to unnecessary antibiotics and H2 blockers in early life. Timely recognition continues to be studied as risk tools are refined but the current state of evidence justifies a “probably do it” recommendation because of the potential for benefit, low risk, and support for consistent communication to strengthen patient safety in other areas. In upcoming years, we anticipate more information will be available to support a broad approach to timely recognition. In the meantime, engaging families and structuring assessments and communication when NEC is suspected could strengthen prompt diagnosis and quick action.

Although we reviewed evidence for holding feeding during blood transfusion, the group did not achieve consensus on best approaches because evidence was inconsistent in 2015. Anemia appears to underlie the risk for NEC with transfusion [117, 118]. Transfusion thresholds differ, few have transfusion protocols in place, and addressing confounders that reduce NEC like feeding protocols and human milk exposiure is not consistent. Experts recommended that if a neonatal group chose to hold feeding during transfusion, they should agree to *how* they will do so, integrate it into the feeding protocol, and address criteria to restart feeding. The approach taken in the multi-site QI project was to hold the feeding only during the transfusion, not advance the feeding volume on the day of the feeding and avoid fortifiers on that day- with significant reduction in the most severe NEC across 8 NICUs [119].

To balance the strengths of this project, we should also address its limitations. Using a scoping review vs. a systematic review approach had the potential to miss important evidence in the literature. We cannot be certain that we included all of the relevant literature. However, we were able to satisfy a diverse group of 20 experts to answer key questions about how to prevent NEC and support its timely recognition using feasible, implementation ready strategies. In an individual NICU’s process to adopt evidence-based interventions this work is typically done by <5 busy clinicians who may find our results helpful to their efforts. We did not engage parents specifically whose children did not get NEC as experts, which may have limited the generalizabilty to all parents. However, the processes useful to prevent NEC are also those that support neonatal health broadly (e.g. avoiding excessive antibiotic exposure, promoting a human milk diet and supporting healthy team communication and risk awareness).

## Conclusion

Stakeholders maintained engagement when they were organized around the task of answering key questions and agreed to actionable, feasible and evidence-based strategies to foster NEC prevention and timely recognition. Implementation strategies addressed staff education, parent engagement, early discussions, structuring communication, integrating reminders into electronic health record systems, and using audit and feedback mechanisms. Partnering national experts with local experts and ensuring that clinical and parent perspectives were sought yielded balanced, focused, and feasible implementation strategies that any NICU could implement today to drive their incidence of NEC to zero.

## Abbreviations

AUC: Area under the curve
CI: Confidence interval
CRP: C-reactive protein
DHM: Donor human milk
FDA: Food and Drug Administration
H2: Histamine-2
HM: Human milk
i-FABP: Intestinal fatty acid-binding protein
NEC: Necrotizing enterocolitis
NICU: Neonatal intensive care unit
NIRS: Near infrared spectroscopy
RCT: Randomized controlled trial
US: United States
VLBW infant: Very low birth weight

## References

[1] Russell RB, Green NS, Steiner CA (2007). Cost of hospitalization for preterm and low birth weight infants in the United States. Pediatrics.

[2] Schulman J, Stricof RL, Stevens TP (2009). Development of a statewide collaborative to decrease NICU central line-associated bloodstream infections. J Perinatol.

[3] Suresh GK, Edwards WH (2012). Central line-associated bloodstream infections in neonatal intensive care: changing the mental model from inevitability to preventability. Am J Perinatol.

[4] Yee WH, Soraisham AS, Shah VS, Aziz K, Yoon W, Lee SK (2012). Incidence and Timing of Presentation of Necrotizing Enterocolitis in Preterm Infants. Pediatrics.

[5] Fanaroff AA, Stoll BJ, Wright LL, et al. Trends in neonatal morbidity and mortality for very low birthweight infants. Am J Obstet Gynecol. 2007;196(2) doi:10.1016/j.ajog.2006.09.014. 10.1016/j.ajog.2006.09.014 17306659

[6] Guner YS, Chokshi N, Petrosyan M, Upperman JS, Ford HR, Grikscheit TC (2008). Necrotizing enterocolitis--bench to bedside: novel and emerging strategies. Semin Pediatr Surg.

[7] Catlin A (2006). Extremely long hospitalizations of newborns in the United States: data, descriptions, dilemmas. J Perinatol.

[8] Rees CM, Eaton S, Pierro A (2008). Trends in infant mortality from necrotising enterocolitis in England and Wales and the USA. Arch Dis Child Fetal Neonatal Ed.

[9] Zhang Y, Ortega G, Camp M, Osen H, Chang DC, Abdullah F (2011). Necrotizing enterocolitis requiring surgery: Outcomes by intestinal location of disease in 4371 infants. J Pediatr Surg.

[10] Luig M, Lui K (2005). Epidemiology of necrotizing enterocolitis - Part II: Risks and susceptibility of premature infants during the surfactant era: A regional study. J Paediatr Child Health.

[11] Horbar JD, Plsek PE, Leahy K (2003). NIC/Q 2000: establishing habits for improvement in neonatal intensive care units. Pediatrics.

[12] Lake ET, Staiger D, Cheung R, Kenny MJ, Patrick T, Rogowski JA (2012). for Nursing Excellence and Outcomes of Very Low-Birth-Weight Infants. Jama J Am Med Assoc.

[13] Fitzgibbons SC, Ching Y, Yu D (2009). Mortality of necrotizing enterocolitis expressed by birth weight categories. J Pediatr Surg.

[14] Lee HC, Martin-Anderson S, Lyndon A, Dudley RA (2013). Perspectives on Promoting Breastmilk Feedings for Premature Infants During a Quality Improvement Project. Breastfeed Med.

[15] Cincinnati Children’s Medical Center. Evidence-Based Care Guideline for Necrotizing Enterocolitis (NEC) among Very Low Birth Weight Infants.; 2010.

[16] Graham R, Mancher M, Miller Wolman D, Greenfield S, Steinberg E, editors. Institute of Medicine (US) Committee on Standards for Developing Trustworthy Clinical Practice. In: Clinical Practice Guidelines We Can Trust. Washington (DC); 2011. doi:10.17226/13058. 24983061

[17] Brouwers MC, Kho ME, Browman GP (2010). AGREE II: advancing guideline development, reporting and evaluation in health care. CMAJ.

[18] Atkins D, Best D, Briss PA (2004). Grading quality of evidence and strength of recommendations. BMJ.

[19] Guyatt GH, Oxman AD, Kunz R (2008). Going from evidence to recommendations. BMJ.

[20] Titler MG, Everett LQ (2001). Translating research into practice. Considerations for critical care investigators. Crit Care Nurs Clin North Am.

[21] Moore JE, Titler MG, Kane Low L, Dalton VK, Sampselle CM (2015). Transforming Patient-Centered Care: Development of the Evidence Informed Decision Making through Engagement Model. Womens Health Issues.

[22] Titler MG. The Evidence for Evidence-Based Practice Implementation. In: Hughes RG, ed. Patient Safety and Quality: An Evidence-Based Handbook for Nurses. Rockville (MD); 2008.

[23] Titler MG (2010). Translation science and context. Res Theory Nurs Pract.

[24] Valente TW, Rogers EM (1995). The origins and development of the diffusion of innovations paradigm as an example of scientific growth. Sci Commun.

[25] Mays N, Roberts E, Popay J. Synthesizing research evidence. In: Fulop N, Allen P, Clarke A, N B, eds. Studying the Organisation and Delivery of Health Services: Research Methods. London: Routledge; 2001:194.

[26] Arksey H, O’Malley L (2005). Scoping studies: towards a methodological framework. Int J Soc Res Methodol.

[27] Levac D, Colquhoun H, O’Brien KK (2010). Scoping studies: advancing the methodology. Implement Sci.

[28] Colquhoun HL, Levac D, O’Brien KK (2014). Scoping reviews: Time for clarity in definition, methods, and reporting. J Clin Epidemiol.

[29] Lucas A, Cole TJ. Breast milk and neonatal necrotising enterocolitis. *Lancet (London, England)*. 1990;336(8730):1519–1523. 10.1016/0140-6736(90)93304-8 1979363

[30] Sullivan S, Schanler RJ, Kim JH, et al. An Exclusively Human Milk-Based Diet Is Associated with a Lower Rate of Necrotizing Enterocolitis than a Diet of Human Milk and Bovine Milk-Based Products. J Pediatr. 2010;156(4) doi:10.1016/j.jpeds.2009.10.040. 10.1016/j.jpeds.2009.10.040 20036378

[31] Schanler RJ (2011). Outcomes of human milk-fed premature infants. Semin Perinatol.

[32] Rodriguez NA, Meier PP, Groer MW, Zeller JM, Engstrom JL, Fogg L (2010). A Pilot Study to Determine the Safety and Feasibility of Oropharyngeal Administration of Own Motherʼs Colostrum to Extremely Low-Birth-Weight Infants. Adv Neonatal Care.

[33] Rodriguez NA, Groer MW, Zeller JM (2011). A randomized controlled trial of the oropharyngeal administration of mother’s colostrum to extremely low birth weight infants in the first days of life. Neonatal Intensive Care.

[34] Lee J, Kim HS, Jung YH (2016). Oropharyngeal colostrum administration in extremely premature infants: An RCT. World Rev Nutr Diet.

[35] Montgomery DP, Baer VL, Lambert DK, Christensen RD (2010). Oropharyngeal administration of colostrum to very low birth weight infants: Results of a feasibility trial. Neonatal Intensive Care.

[36] Seigel JK, Smith PB, Ashley PL (2013). Early Administration of Oropharyngeal Colostrum to Extremely Low Birth Weight Infants. Breastfeed Med.

[37] Gephart SM, Hanson CK (2013). Preventing Necrotizing Enterocolitis With Standardized Feeding Protocols. Adv Neonatal Care..

[38] Patole SK (2005). Impact of standardised feeding regimens on incidence of neonatal necrotising enterocolitis: a systematic review and meta-analysis of observational studies. Arch Dis Child - Fetal Neonatal Ed.

[39] Cotten CM, Taylor S, Stoll B (2009). Prolonged Duration of Initial Empirical Antibiotic Treatment Is Associated With Increased Rates of Necrotizing Enterocolitis and Death for Extremely Low Birth Weight Infants. Pediatrics.

[40] Alexander VN, Northrup V, Bizzarro MJ (2011). Antibiotic exposure in the newborn intensive care unit and the risk of necrotizing enterocolitis. J Pediatr.

[41] Guillet R, Stoll BJ, Cotten CM (2006). Association of H2-Blocker Therapy and Higher Incidence of Necrotizing Enterocolitis in Very Low Birth Weight Infants. Pediatrics.

[42] Terrin G, Passariello A, De Curtis M (2012). Ranitidine is Associated With Infections, Necrotizing Enterocolitis, and Fatal Outcome in Newborns. Pediatrics.

[43] Gantz M, Roy J, Guillet R (2008). Analyzing retrospective data with time-varying exposure: A cautionary tale of H2 blockers in ELBW neonates. Am J Perinatol.

[44] Gephart SM, Spitzer AR, Effken JA, Dodd E, Halpern M, McGrath JM (2014). Discrimination of GutCheckNEC: a clinical risk index for necrotizing enterocolitis. J Perinatol.

[45] Gephart SM, Wetzel C, Krisman B (2014). Prevention and Early Recognition of Necrotizing Enterocolitis. Adv Neonatal Care..

[46] Gephart SM (2012). Transfusion-Associated Necrotizing Enterocolitis. Adv Neonatal Care..

[47] Christensen RD (2011). Association between red blood cell transfusions and necrotizing enterocolitis. J Pediatr.

[48] Mohamed A, Shah PS (2012). Transfusion Associated Necrotizing Enterocolitis: A Meta-analysis of Observational Data. Pediatrics.

[49] Alfaleh K, Anabrees J, Bassler D (2011). Probiotics for prevention of necrotizing enterocolitis in preterm infants ( Review ). Cochrane.

[50] Thakkar HS, Lakhoo K (2016). Necrotizing enterocolitis. Surg.

[51] Guyatt G, Oxman AD, Akl EA (2011). GRADE guidelines: 1. Introduction - GRADE evidence profiles and summary of findings tables. J Clin Epidemiol.

[52] Owens DK, Lohr KN, Atkins D (2010). AHRQ series paper 5: grading the strength of a body of evidence when comparing medical interventions--agency for healthcare research and quality and the effective health-care program. J Clin Epidemiol.

[53] Andrews J, Guyatt G, Oxman AD (2013). GRADE guidelines: 14. Going from evidence to recommendations: The significance and presentation of recommendations. J Clin Epidemiol.

[54] Spatz D, Edwards T (2015). The use of human milk and breastfeeding in the neonatal intensive care unit: NANN position statement #3052. Adv neonatal care.

[55] American Academy of Pediatrics (2012). Breastfeeding and the Use of Human Milk. Pediatrics.

[56] Association of Women’s Health O and NN (2015). AWHONN Position Statement: Breastfeeding. J Obstet Gynecol Neonatal Nurs.

[57] WHO/UNICEF. Global Nutrion Target 2025. Breastfeeding policy brief. WHO/MNH/NHD 14.7. WHO Libr Cat Data. 2014:8. doi:WHO/NMH/NHD/14.7.

[58] Q M (2014). Formula versus donor breast milk for feeding preterm or low birth weight infants. Cochrane database Syst Rev.

[59] Marx C, Bridge R, Wolf AK, Rich W, Kim JH, Bode L (2014). Human milk oligosaccharide composition differs between donor milk and mother’s own milk in the NICU. J Hum Lact.

[60] Meier PP, Johnson TJ, Patel AL, Rossman B (2017). Evidence-Based Methods That Promote Human Milk Feeding of Preterm Infants: An Expert Review. Clin Perinatol.

[61] Cristofalo EA, Schanler RJ, Blanco CL (2013). Randomized trial of exclusive human milk versus preterm formula diets in extremely premature infants. J Pediatr.

[62] Herrmann K, Carroll K (2014). An Exclusively Human Milk Diet Reduces Necrotizing Enterocolitis. Breastfeed Med.

[63] Assad M, Elliott MJ, Abraham JH (2016). Decreased cost and improved feeding tolerance in VLBW infants fed an exclusive human milk diet. J Perinatol.

[64] Abrams SA, Schanler RJ, Lee ML, Rechtman DJ (2014). Greater mortality and morbidity in extremely preterm infants fed a diet containing cow milk protein products. Breastfeed Med.

[65] Hair AB, Hawthorne KM, Chetta KE, Abrams SA (2013). Human milk feeding supports adequate growth in infants ≤ 1250 grams birth weight. BMC Res Notes.

[66] Gephart SM, Weller M (2014). Colostrum as Oral Immune Therapy to Promote Neonatal Health. Adv Neonatal Care..

[67] Romano-Keeler J, Azcarate-Peril MA, Weitkamp J-H (2017). Oral colostrum priming shortens hospitalization without changing the immunomicrobial milieu. J Perinatol.

[68] Lee J, Kim H-S, Young HJ (2015). Oropharyngeal Colostrum Administration in Extremely Premature Infants: An RCT. Pediatrics.

[69] Thibeau S, Boudreaux C (2013). Exploring the use of mothers’ own milk as oral care for mechanically ventilated very low-birth-weight preterm infants. Adv Neonatal Care..

[70] Froh EB, Deatrick JA, Curley MAQ, Spatz DL (2015). Making Meaning of Pumping for Mothers of Infants With Congenital Diaphragmatic Hernia. J Obstet Gynecol Neonatal Nurs.

[71] Rodriguez NA, Vento M, Claud EC, Wang CE, Caplan MS (2015). Oropharyngeal administration of mother’s colostrum, health outcomes of premature infants: study protocol for a randomized controlled trial. Trials.

[72] Colaizy TT, Bartick MC, Jegier BJ (2016). Impact of Optimized Breastfeeding on the Costs of Necrotizing Enterocolitis in Extremely Low Birthweight Infants. J Pediatr.

[73] Spatz DL (2004). Ten steps for promoting and protecting breastfeeding for vulnerable infants. J Perinat Neonatal Nurs..

[74] Kim JH, Chan CS, Vaucher YE, Stellwagen LM (2013). Challenges in the practice of human milk nutrition in the neonatal intensive care unit. Early Hum Dev.

[75] Meier PP, Engstrom JL, Mingolelli SS, Miracle DJ, Kiesling S (2004). The Rush Mothers’ Milk Club: breastfeeding interventions for mothers with very-low-birth-weight infants. J Obstet Gynecol neonatal Nurs JOGNN.

[76] Bingham EM (2012). Optimizing nutrition in the neonatal intensive care unit: a look at enteral nutrition and the prevention of necrotizing enterocolitis. Top Clin Nutr.

[77] Dutta S, Singh B, Chessell L (2015). Guidelines for feeding very low birth weight infants. Nutrients.

[78] Berseth CL, Bisquera JA, Paje VU (2003). Prolonging small feeding volumes early in life decreases the incidence of necrotizing enterocolitis in very low birth weight infants. Pediatrics.

[79] Morgan J, Bombell S, McGuire W (2013). Early trophic feeding versus enteral fasting for very preterm or very low birth weight infants. Cochrane Database Syst Rev.

[80] Morgan J, Young L, McGuire W (2015). Slow advancement of enteral feed volumes to prevent necrotising enterocolitis in very low birth weight infants. Cochrane Database Syst Rev.

[81] Hanson C, Sundermeier J, Dugick L, Lyden E, Anderson-Berry AL (2011). Implementation, Process, and Outcomes of Nutrition Best Practices for Infants &lt;1500 g. Nutr Clin Pract.

[82] Smith JR (2005). Early enteral feeding for the very low birth weight infant: the development and impact of a research-based guideline. Neonatal Netw.

[83] McCallie KR, Lee HC, Mayer O, Cohen RS, Hintz SR, Rhine WD (2011). Improved outcomes with a standardized feeding protocol for very low birth weight infants. J Perinatol.

[84] Viswanathan S, McNelis K, Super D, Einstadter D, Groh-Wargo S, Collin M (2015). Standardized Slow Enteral Feeding Protocol and the Incidence of Necrotizing Enterocolitis in Extremely Low Birth Weight Infants. J Parenter Enter Nutr.

[85] Butler TJ, Szekely LJ, Grow JL (2013). A standardized nutrition approach for very low birth weight neonates improves outcomes, reduces cost and is not associated with increased rates of necrotizing enterocolitis, sepsis or mortality. J Perinatol.

[86] Loomis T, Byham-Gray L, Ziegler J, Parrott JS (2014). Impact of Standardized Feeding Guidelines on Enteral Nutrition Administration, Growth Outcomes, Metabolic Bone Disease, and Cholestasis in the NICU. J Pediatr Gastroenterol Nutr.

[87] Street JL, Montgomery D, Alder SC, Lambert DK, Gerstmann DR, Christensen RD. Implementing feeding guidelines for NICU patients < 2000 g results in less variability in nutrition outcomes. J Parenter Enter Nutr. 2006;30(6):515–518. doi:30/6/515. 10.1177/0148607106030006515 17047177

[88] Patole SKK, Kadalraja R, Tuladhar R, Almonte R, Muller R, Whitehall JSS (2000). Benefits of a standardised feeding regimen during a clinical trial in preterm neonates. Int J Clin Pract.

[89] Premji SS (2002). A Matched Cohort Study of Feeding Practice Guidelines for Infants Weighing Less Than 1,500 g. Adv Neonatal Care..

[90] Kamitsuka MD, Horton MK, Williams MA (2000). The incidence of necrotizing enterocolitis after introducing standardized feeding schedules for infants between 1250 and 2500 grams and less than 35 weeks of gestation. Pediatrics.

[91] Kuzma-O’Reilly B, Duenas ML, Greecher C (2003). Evaluation, Development and Implementation of Potentially Better Practices in Neonatal Intensive Care Nutrition. Pediatrics.

[92] Patole S, McGlone L, Muller R (2003). Virtual elimination of necrotising enterocolitis for 5 years - Reasons?. Med Hypotheses.

[93] Collaborative CPQ. CPQCC Nutrition Toolkit Appendices.; 2008. https://www.cpqcc.org/qi-tool-kits/nutritional-support-vlbw-infant.

[94] Lee Chong H, Kurtin PS, Wight NE (2012). A Quality Improvement Project to Increase Breast Milk Use in Very Low Birth Weight Infants. Pediatrics.

[95] Gephart SM, McGrath JM, Effken JA, Halpern MD (2012). Necrotizing Enterocolitis Risk. Adv Neonatal Care..

[96] Gregory KE. Disease Prediction Strategies for Necrotizing Enterocolitis. J Perinat Neonatal Nurs. 2015:5–7. doi:10.1097/JPN.0000000000000088. 10.1097/JPN.0000000000000088 25633394

[97] Nantais-Smith L, Kadrofske M (2015). Noninvasive biomarkers of necrotizing enterocolitis. J Perinat Neonatal Nurs..

[98] Patel AK, Lazar DA, Burrin DG (2014). Abdominal Near-Infrared Spectroscopy Measurements Are Lower in Preterm Infants at Risk for Necrotizing Enterocolitis. Pediatr Crit Care Med.

[99] Marin T, Moore J, Kosmetatos N (2013). Red blood cell transfusion-related necrotizing enterocolitis in very-low-birthweight infants: A near-infrared spectroscopy investigation. Transfusion.

[100] Marin T, Josephson CD, Kosmetatos N, Higgins M, Moore JE (2014). Feeding preterm infants during red blood cell transfusion is associated with a decline in postprandial mesenteric oxygenation. J Pediatr.

[101] Naberhuis J, Wetzel C, Tappenden KA. A Novel Neonatal Feeding Intolerance and Necrotizing Enterocolitis Risk-Scoring Tool Is Easy to Use and Valued by Nursing Staff. Adv Neonatal Care. 2016;16(3):239–44. doi:10.1097/ANC.0000000000000250 10.1097/ANC.0000000000000250 26825014

[102] Fox JR, Thacker LR, Hendricks-Muñoz KD (2015). Early Detection Tool of Intestinal Dysfunction: Impact on Necrotizing Enterocolitis Severity. Am J Perinatol.

[103] Gephart SM, Cholette M (2012). PURE Communication: A Strategy to Improve Care Coordination for High-Risk Birth. Newborn Infant Nurs Rev.

[104] Gephart SM, McGrath JM, Effken JA (2011). Failure to rescue in neonatal care. J Perinat Neonatal Nurs.

[105] Brady PW, Muething S, Kotagal U (2013). Improving situation awareness to reduce unrecognized clinical deterioration and serious safety events. Pediatrics.

[106] Canvasser J, Gadepelli S, Gephart S, Kim J. International Survey of Parental Perspectives of NEC Communication. In: E-PAS. San Diego, CA: Pediatric Academic Societies; 2015:2905.459.

[107] Kuppala VS, Meinzen-Derr J, Morrow AL, Schibler KR (2011). Prolonged initial empirical antibiotic treatment is associated with adverse outcomes in premature infants. J Pediatr.

[108] Krediet T, Lelyveld N, Vijlbrief D (2007). Microbiological factors associated with neonatal necrotizing enterocolitis: protective effect of early antibiotic treatment. Acta Paediatr.

[109] Tagare A, Kadam S, Vaidya U, Pandit A (2010). Routine antibiotic use in preterm neonates: A randomised controlled trial. J Hosp Infect.

[110] Squire E, Favara B, Todd J (1979). Diagnosis of neonatal bacterial infection: Hematologic and pathologic findings in fatal and nonfatal cases. Pediatrics.

[111] More K, Athalye-Jape G, Rao S, Patole S (2013). Association of Inhibitors of Gastric Acid Secretion and Higher Incidence of Necrotizing Enterocolitis in Preterm Very Low-Birth-Weight Infants. Am J Perinatol.

[112] Patel SJ, Saiman L, Duchon JM, Evans D, Ferng YH, Larson E. Development of an antimicrobial stewardship intervention using a model of actionable feedback. Interdiscip Perspect Infect Dis. 2012;2012(c) doi:10.1155/2012/150367. 10.1155/2012/150367 PMC3303556 22500166

[113] Polin RA (2012). Management of Neonates With Suspected or Proven Early-Onset Bacterial Sepsis. Pediatrics.

[114] Mukhopadhyay S, Puopolo KM (2017). Clinical and Microbiologic Characteristics of Early-onset Sepsis Among Very Low Birth Weight Infants: Opportunities for Antibiotic Stewardship. Pediatr Infect Dis J.

[115] Puopolo KM, Draper D, Wi S (2011). Estimating the probability of neonatal early-onset infection on the basis of maternal risk factors. Pediatrics.

[116] Escobar GJ, Puopolo KM, Wi S (2014). Stratification of Risk of Early-Onset Sepsis in Newborns &gt;=34 Weeks’ Gestation. Pediatrics.

[117] Singh R, Visintainer PF (2011). Association of necrotizing enterocolitis with anemia and packed red blood cell transfusions in preterm infants. J Perinatol.

[118] Patel RM, Knezevic A, Shenvi N (2016). Association of Red Blood Cell Transfusion, Anemia, and Necrotizing Enterocolitis in Very Low-Birth-Weight Infants. JAMA.

[119] Talavera MM, Bixler G, Cozzi C, et al. Quality Improvement Initiative to Reduce the Necrotizing Enterocolitis Rate in Premature Infants. Pediatrics. 2016;137(5) doi:10.1542/peds.2015-1119. 10.1542/peds.2015-1119 27244778

